# Adding Amino Acids to a Sucrose Diet Is Not Sufficient to Support Longevity of Adult Bumble Bees

**DOI:** 10.3390/insects11040247

**Published:** 2020-04-15

**Authors:** Nils Grund-Mueller, Fabian A. Ruedenauer, Johannes Spaethe, Sara D. Leonhardt

**Affiliations:** 1 Department of Animal Ecology and Tropical Biology (Zoology III), University of Würzburg, 97074 Würzburg, Germany; nils.schmucker@stud-mail.uni-wuerzburg.de (N.G.-M.); fabian.ruedenauer@tum.de (F.A.R.); leonhardt@wzw.tum.de (S.D.L.); 2 Plant-Insect Interactions Group, Technical University of Munich, 85354 Freising, Germany; 3 Department of Behavioral Physiology and Sociobiology (Zoology II), University of Würzburg, 97074 Würzburg, Germany

**Keywords:** nutrition, nutrients, foraging, pollen, resources, adult bees

## Abstract

Dietary macro-nutrients (i.e., carbohydrates, protein, and fat) are important for bee larval development and, thus, colony health and fitness. To which extent different diets (varying in macro-nutrient composition) affect adult bees and whether they can thrive on nectar as the sole amino acid source has, however, been little investigated. We investigated how diets varying in protein concentration and overall nutrient composition affected consumption, longevity, and breeding behavior of the buff-tailed bumble bee, *Bombus terrestris* (Hymenoptera: Apidae). Queenless micro-colonies were fed either natural nutrient sources (pollen), nearly pure protein (i.e., the milk protein casein), or sucrose solutions with low and with high essential amino acid content in concentrations as can be found in nectar. We observed micro-colonies for 110 days. We found that longevity was highest for pure pollen and lowest for pure sucrose solution and sucrose solution supplemented with amino acids in concentrations as found in the nectar of several plant species. Adding higher concentrations of amino acids to sucrose solution did only slightly increase longevity compared to sucrose alone. Consequently, sucrose solution with the applied concentrations and proportions of amino acids or other protein sources (e.g., casein) alone did not meet the nutritional needs of healthy adult bumble bees. In fact, longevity was highest and reproduction only successful in micro-colonies fed pollen. These results indicate that, in addition to carbohydrates and protein, adult bumble bees, like larvae, need further nutrients (e.g., lipids and micro-nutrients) for their well-being. An appropriate nutritional composition seemed to be best provided by floral pollen, suggesting that pollen is an essential dietary component not only for larvae but also for adult bees.

## 1. Introduction

An insect’s stress tolerance and survival depend, among other things, on the amount and ratio of particular nutrients consumed [1,2,3,4]. Thus, food source quality and availability play a major role in insect survival, as they directly determine the nutritional composition of the diets [5,6]. 

The diet of bees is usually comprised of nectar and pollen [7,8]. Nectar primarily contains carbohydrates, but also low amounts of protein, free amino acids, lipids, and phytochemicals [9,10,11]. Nectar is considered the main food source of adult bees [12]. Pollen, in turn, is predominantly consumed by larvae and represents the primary source of protein and fat [7,13,14]. Pollen is further comprised of carbohydrates, vitamins, sterols, carotenoids, and flavonoids [15,16], rendering pollen an important source of various nutrients.

As bees are thought to consume pollen primarily during larval growth and female egg production [12,17,18], it was assumed that pollen, in contrast to nectar, does not seem to play a major nutritional role for adult bees [12]. However, recent studies in honey bees (*Apis mellifera*) and bumble bees (*Bombus impatiens*) showed that longevity of adult bees depended on the protein-to-carbohydrate [19,20,21] or protein-to-lipid ratio [4,22] of food consumed and decreased with increasing (excess) protein content or protein-to-carbohydrate ratio [23,24]. On the other hand, diets of high (non-excess) protein content positively affected the immune response against pathogens in adult honey bees [19,25,26,27,28] and increased reproduction [29,30] and survival [29,31], suggesting that, besides carbohydrates, protein may also have an impact on adult bees. 

While many studies focused on short-term diet effects and observed colonies for only a few days (e.g., one to two weeks [e.g., 22,23]), our goal was to investigate the effect of different diets (i.e., different protein and amino acid sources) on food consumption, longevity, and reproduction of adult *B. terrestris* workers over longer periods. We observed workers kept in queenless micro-colonies for 110 days. The effect of diet on queenless micro-colonies was comparable to effects on queenright macro-colonies, as has been shown by Génissel, Aupinel, Bressac, Tasei, and Chevrier [29] and Smeets and Duchateau [31]. We tested diets that differed in nutritional composition of protein, i.e., (1) commercial pollen blends as natural nutrient source, (2) the milk protein casein (which has been used in the past as protein substitute for bees [e.g., 20,32]) as nearly pure protein source, and (3) pure amino acids added to a sucrose solution, mimicking the amino acid content of nectar. 

As nectar is considered the primary food source of adult bumble bees, we hypothesized that amino acid concentrations as found in nectar are sufficient to increase longevity (compared to pure sucrose). Given the positive effect of pollen protein content on adult bee immunity and breeding performance shown by previous studies (see above), we further expected that longevity and reproductive success increased for diets containing some sort of protein even in relatively low concentration. Because pollen contains other nutrients in addition to protein, we expected that longevity was highest when fed pollen and slightly reduced when fed casein, which lacks such additional nutrients.

## 2. Material and Methods

### 2.1. Experimental Setup

The experiments were conducted at the University of Würzburg from May to December 2014. We tested 25 micro-colonies, each with ten *Bombus terrestris* workers, which were randomly composed from five commercial queenright colonies (Koppert, The Netherlands) to ensure that each colony comprised workers of different age and origin and to exclude potential colony-specific pathogen effects. Workers were placed in wooden boxes (14.5 × 13.0 × 10.0 cm), which were thoroughly cleaned with soap, water, and ethanol before usage, and had ad libitum access to a 60% (weight by weight (w/w)) sucrose solution provided in two plastic feeders. Micro-colonies were shown to be comparable to queenright colonies in terms of nutritional intake and reproductive behavior and, therefore, in their nutritional requirements [29]. Workers in micro-colonies typically start to lay unfertilized eggs after a few days, which develop into males. Each of five micro-colonies received one out of five diets (resulting in five micro-colonies for each of the five diets). Different diets were provided on petri dishes and composed of (1) either pure bee-collected pollen (Ppure, Naturwaren-Niederrhein GmbH, Germany; see Table S1 for amino acid composition), (2) casein (Sigma Aldrich, Germany) mixed with sucrose (Csuc, mimicking pollen), (3) pure 60% (by mass) sucrose solution (sucrose), or 60% sucrose solution with 10 essential amino acids and proline (Sigma Aldrich, Germany) in (4) high (AAhigh) or (5) low (AAlow) concentration to mimic a nectar-like diet. Casein is a protein powder obtained from milk, which contains all 20 proteinogenic amino acids and is frequently used in studies on bee nutrition and, therefore, can be used as pollen surrogate [20,24,32,33]. We added sugar and water to the casein diet to create a similar texture as obtained for pollen paste. Amino acid amounts used for the low concentration mixture were the average concentrations found for essential amino acids (which cannot be synthesized by the bees themselves [34]) and proline (a non-essential amino acid which was added because it is important for flight muscle activity in bees [35]) in floral nectar of eight bee-pollinated Fabaceae plants ([36], Table S2). The high concentration mixture contained a hundred-fold higher concentration of each amino acid compared to the natural concentration.

### 2.2. Diet Preparation

To remove sugar (controlled for with Fehling’s test) from bee-collected pollen, and thus better control for total sugar consumption, 200 g of pollen were ground, diluted in 600 mL de-ionized water for 24 h, and filtered. The procedure was repeated with ethanol and the pollen finally dried at 30 °C for 24 h. Pollen was then mixed with de-ionized water in a ratio of 1:1 until it became a viscid mass. Casein was mixed with sucrose (1:3, i.e., one volume equivalent (VE) of casein and three VEs of sucrose) and water to obtain a food mixture that was readily accepted and collected by bees, because they did not forage on pure casein powder mixed with water in preliminary experiments. 

### 2.3. Amino Acid Analysis

For each diet, we ground all components with a mortar to produce a homogenous food slurry. The slurry was mixed with de-ionized water to facilitate stirring and then dried at 30 °C for 24 h. The protein content (i.e., sum of free and protein-bound amino acids) of each of these diets was determined by ion-exchange chromatography ((IEC), Biochrom 20 plus amino acid analyzer, Laborservice Onken, Gründau, Germany) [37,38]. 

In a first step, free amino acids were dissolved in 100 µL of deionized water using an ultrasonic bath (Emmi 20HC, EMAG, Mörfelden-Walldorf, Germany). The resulting extract was kept at 7 °C for 60 min and subsequently membrane filtered for 10 min. It was boiled for 2 min at 100 °C, cooled down to room temperature, and centrifuged for 5 min. The supernatant was mixed with 10 µL of 12.5% 5-sulfosalicylic acid (Merck, Darmstadt, Germany) and kept at 7 °C for another 30 min. It was then centrifuged for 10 min and 50 µL of the supernatant was mixed with 50 µL buffer (Laborservice Onken GmbH, Gründau, Germany). The extract was filtered again and then analyzed by IEC. 

The residue of the membrane filtration in step 1 was hydrolyzed with 200 µL of 6 N HCl to obtain protein-bound amino acids. After boiling for 4 h, the mixture was centrifuged for 10 min. Water was evaporated and the sample was resolved in 200 mL deionized water and centrifuged thrice. Then, 100 µL of the supernatant was mixed with 20 µL of 12.5% sulfosalicylic acid and refrigerated for 30 min. After centrifuging for 10 min, 100 µL of the supernatant was mixed with 100 µL buffer and filtered. The filtrate was analyzed by IEC. With this procedure, all proteinogenic amino acids besides tryptophan can be detected. Since tryptophan concentrations are highly variable in nectar [39,40,41,42], we estimated a plausible concentration for tryptophan by calculating the mean concentration of all other amino acids. 

### 2.4. Data Acquisition

All diets were provided fresh and ad libitum every day. Consumption of diets was recorded daily by weighing each dish before placing it inside boxes and again the next day. To obtain mean individual consumption, amounts consumed were divided by the number of individuals present when removing the dish. To account for evaporation, we placed control dishes of each diet next to our boxes and measured their weight loss every 24 h. We had measured evaporation rates with different methods for a previous study [5] and found that evaporation was similar for dishes placed inside a colony (i.e., box) and dishes placed outside. The values for evaporation were then used to correct our consumption records by subtracting the daily weight lost due to evaporation. Likewise, consumption of sucrose solution per individual was obtained by dividing the overall amount of sucrose solution consumed within one day by the number of individuals present. In addition, we recorded longevity by counting the number of dead bees per day and scored successful breeding behavior (i.e., rearing of larvae to adult drones) every day. Workers that died in the first week were replaced with new workers from the original colonies, as we attributed their death to experimental stress or age, but not diet. Bees were replaced initially because they are more likely to start breeding in relatively larger worker groups. Freshly hatched drones were removed daily and frozen to prevent any bias of food consumption. Colonies were observed for a total of 110 days.

### 2.5. Statistical Analysis

All analyses were conducted in R (version 3.1.1) [43].

To analyze differences in daily diet (overall food, protein, and sucrose) consumption per individual, the amount of food each individual consumed from each diet was averaged over the number of days the colony survived. Exact amounts of protein consumed were determined based on the mass proportion of pollen/casein fed and the protein concentrations (i.e., sum of all amino acids) obtained by the IEC analysis. Differences in overall food, sucrose, and protein consumption (in mg) were examined by analyses of variance (ANOVA) followed by Tukey tests for post hoc analyses. All data were tested for normality (using Shapiro test) and homogeneity of variances (Bartlett test) and were log- or square root-transformed when necessary. If one or both criteria were not met (even after transformation), we used nonparametric tests instead (i.e., Kruskal–Wallis-tests and pairwise Wilcoxon–Mann–Whitney-tests or Spearman rank correlations).

Differences in longevity of individuals fed different diets were analyzed with Kaplan–Meier statistics (log-rank test, R-packages: survival & KMsurv). The Bonferroni method was used to correct for potential type I errors due to (1) data dependency as a consequence of using the same bee individuals for the consumption and survival analysis and (2) multiple testing when comparing single diets for survival.

## 3. Results

### 3.1. Consumption

**Figure 1:**
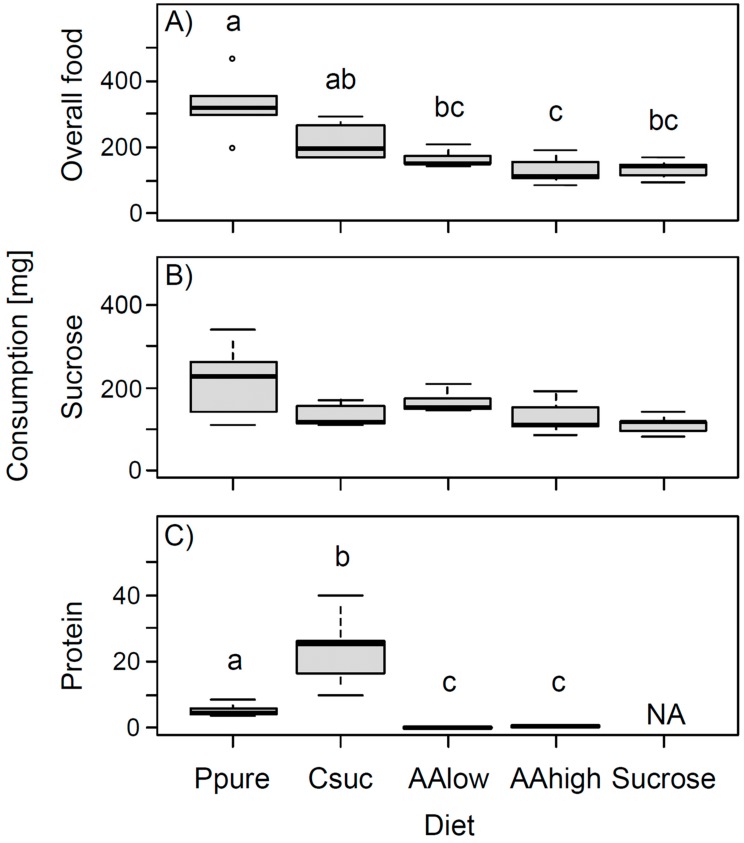
Daily individual consumption (in mg) of (**A**) overall food (including food consumed from petri dishes and from 60% sucrose solutions (with or without amino acids)), (**B**) sucrose (from the 60% sucrose solution plus sucrose from the casein-sucrose mixture), and (**C**) protein (from either pollen, casein, or amino acid mixtures). Different letters indicate significant differences between diets based on Tukey post hoc tests for analyses significant after Bonferroni correction. Abbreviations as follows: Ppure, pure pollen; Csuc, casein-sucrose; AAlow, 60% sucrose solution with concentrations of essential amino acids and proline as can be found in some Fabaceae species; AAhigh, 60% sucrose solution with amino acids at a 100 times higher concentration; Sucrose = only sucrose solution (60% by mass); NA, no data available.

Daily individual overall food consumption (total amount of food consumed in mg, including food consumed from petri dishes and from the 60% sucrose solution) differed significantly between different diets (ANOVA: *F* = 10.64, *p* < 0.001, significance level after Bonferroni: α = 0.0125, Figure 1A). Bumble bees fed pure pollen consumed the highest amount of food, while bumble bees receiving only sucrose solution or sucrose solution with amino acids consumed the lowest amounts (Figure 1A). 

Daily individual overall consumption of sucrose was not significantly different between diets after Bonferroni correction (ANOVA: *F* = 3.42, *p* = 0.028, Figure 1B). However, bumble bees consumed more than two times more sucrose when additionally fed pure pollen than when fed pure sucrose solution only (Figure 1B).

Treatment groups further differed in the amount of protein consumed per individual and day (ANOVA: *F* = 114.80, *p* < 0.001, Figure 1C). Most protein was consumed by bees fed the casein-sucrose mixture followed by bees fed pure pollen (Figure 1C). 

### 3.2. Longevity

**Figure 2:**
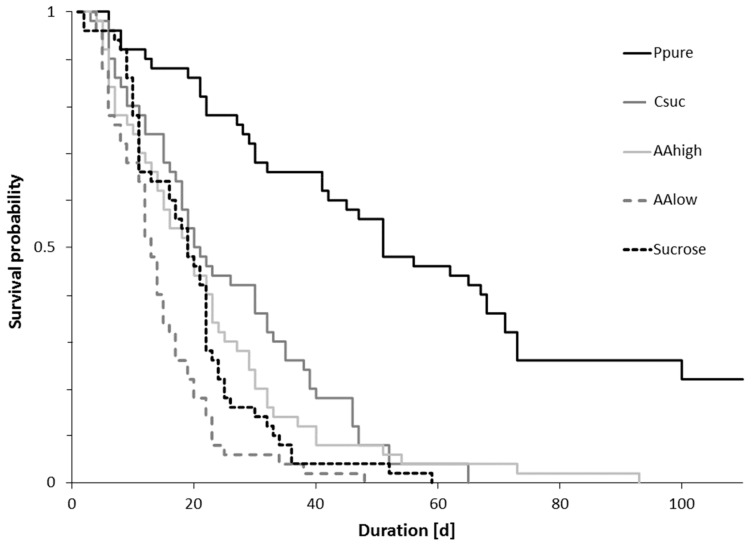
Survival probability of bumble bees (N = 50 per treatment) kept in micro-colonies fed pure pollen (Ppure), casein-sucrose (1:3, Csuc), only sucrose solution (60% by mass, Sucrose), 60% sucrose solution with concentrations of essential amino acids and proline (AAlow), and 60% sucrose solution with amino acids at a 100 times higher concentration (AAhigh).

Overall, longevity of bumble bee workers in micro-colonies differed between diets (log-rank test: *χ^2^* = 186, *p* < 0.001, Figure 2). It was highest for bees fed pure pollen and lowest in colonies fed pure sucrose solution and sucrose solution with low concentrations of amino acids (Figure 2, Table 1). 

Median survival time (Table 2) of bumble bee workers significantly differed between treatments (ANOVA: *F* = 16.61, *p* = 0.001). Only micro-colonies consuming the pure pollen diet successfully raised brood, whereas larvae in colonies consuming other diets died after 2–4 days (data not shown). 

## 4. Discussion

Adult longevity, and thus survival, was strongly influenced by diet in *Bombus terrestris* workers. As expected, longevity was low when bees were fed only sucrose solution, as has also been shown by Smeets and Duchateau [31]. However, longevity was also low when bees were fed artificial diets containing protein (casein) or amino acids, including “artificial nectar” with average amino acid concentrations as can be found in some Fabaceae plants (see Table S2 for species list). This finding disagreed with our hypothesis and previous assumptions (e.g., [31]) that longevity increases when amino acids or protein are provided in addition to sucrose. It is possible, however, that neither casein nor our “artificial nectar” provided appropriate ratios of (essential) amino acids, even though at least casein contained all 20 proteinogenic amino acids [24,44]. 

Nevertheless, compared to pure sucrose solution or sucrose solution enriched with amino acids, adult bees lived longer when fed the casein-sucrose mixture. Such a positive effect of casein on longevity was also found by Altaye, Pirk, Crewe, and Nicolson [20] for honey bees. In our study, longevity was highest for adult bumble bees fed pure pollen, which indicates that not only larvae, but also adult bees need additional nutrients, which are typically found in pollen besides carbohydrates and protein, to maximize life span.

Animal fitness depends on food of a species-specific nutrient ratio [45]. Because this optimal ratio is rarely ever found in nature, animals must frequently choose between over- or undereating one nutrient to consume sufficient amounts of another [45]. Some studies showed that there can be a trade-off between longevity and reproduction, caused by the amount of consumed protein, as seen e.g., in *Drosophila* [46,47], where high protein content led to increased reproductive success, but reduced longevity. Moreover, some free amino acids can be toxic even at low concentrations [48]. The positive effect of protein can, therefore, become negative if it is consumed in excess amounts or if a protein source contains certain amino acids in detrimentally high concentrations. Interestingly, the bees in our study consumed similar amounts of carbohydrates per diet, suggesting that they regulated carbohydrate rather than amino acids or protein intake. This finding is surprising because *B. terrestris* workers can taste some amino acids dissolved in water as well as differences in their concentrations [49]. They should, therefore, be able to also taste them in the artificial nectar diets used in our study, which is a prerequisite for regulation of their intake. The lack of amino acid regulation suggested by this study’s findings does, however, agree with another study from our group, which also found no regulation of amino acid intake in *B. terrestris* foraging on pollen [50], suggesting that *B. terrestris* may instead regulate other nutrients, such as lipids [50] or carbohydrates. 

The regulation of carbohydrates instead of amino acids could in turn have resulted in the bees overeating amino acids. Previous studies also found that honey bees and bumble bees overeat protein to obtain sufficient carbohydrates when offered a diet with a sub-optimal protein-to-carbohydrate ratio (i.e., more protein and less carbohydrate than would be optimal), which decreased their longevity [23,24]. The bumble bees in our study had, however, ad libitum access to carbohydrates (i.e., a 60% sucrose solution) and were, therefore, not forced to overeat protein to obtain sufficient carbohydrates. They may, however, have overeaten protein to obtain sufficient lipids or a suitable protein-lipid ratio, at least when fed the casein diet (according to the manufacturer, lipids are the main other component of casein), as indicated by the high protein consumption on this diet. In fact, lipids appear to be more strongly regulated by foraging bumble bees than carbohydrates [4,22,51] and may significantly affect colony [4,52,53] and also individual health [50]. Alternatively, the *B. terrestris* workers in our study may simply have ignored amino acids and may have focused on sugar intake only. If the bees indeed regulated carbohydrates/sugar, the nectar-like diets might not have provided sufficient amino acids (or other nutrients, which we did not provide), resulting in reduced longevity.

In contrast to casein, pollen contains much lower protein proportions, a comparably higher fat content and additional nutrients, such as sterols, vitamins, and lipids [14,15,16], which most likely explains why adult bumble bees performed best when fed pollen (in addition to sucrose solution). Génissel, Aupinel, Bressac, Tasei, and Chevrier [29] further suggested that ovary formation in bumble bees most likely depends not only on protein access, but also on the availability of sterols and lipids, which may explain why, in our study, only bumble bees with access to pollen could successfully raise brood. This likely led to a higher pollen consumption and prevented us from comparing reproductive success between different treatments. The lack of these nutrients may also have contributed to the lower survival in those micro-colonies that did not receive any pollen. In fact, sterols and lipids are known to positively affect longevity (e.g., [54]) and reproduction [54,55,56,57,58] in insects. Hymenopterans cannot synthesize sterols themselves and, therefore, need phytosterols to synthesize, e.g., molting hormones [56,59]. Bees can obtain sterols only from floral pollen, which may explain why bumble bees tend to collect pollen of high phytosterol content [60]. In contrast to sterols, vitamins appear not to be essential for brood rearing in honey bees [61]. If and how they affect longevity in honey bees and bumble bees is however still unknown.

## 5. Conclusions

Our results demonstrated that adult *B. terrestris* workers, which have access to nutrients other than sucrose and pure protein, as provided by pollen, can live significantly longer than bees fed diets containing only sucrose, sucrose and casein, or sucrose refined by a specific concentration and composition of amino acids. It remains to be investigated, however, how different proportions and ratios of amino acids, as found in nectar of different plant groups, affect worker longevity. In our study, workers further needed pollen to successfully rear male brood, indicating that healthy *B. terrestris* adults require the same spectrum (albeit different ratios) of micro- and macro-nutrients as larvae in order to thrive and reproduce. Future experiments should aim to better understand the underlying factors explaining reduced survival of bees that consumed inappropriate diets. For example, bee survival could be impaired by physiological or pathological effects of diets, as found in other studies investigating effects of malnutrition in bees (e.g., [19,21,62]).

## Figures and Tables

**Table 1:** Differences in longevity of bumble bees kept in micro-colonies fed different diets. *P*-values <0.05 are marked in bold. *P*-values that are significant after Bonferroni correction are additionally marked in italics. Abbreviations as follows: Pure pollen (Ppure), casein-sucrose (Csuc), only sucrose solution (60% by mass, Sucrose), 60% sucrose solution with natural concentrations of essential amino acids and proline (AAlow), and 60% sucrose solution with amino acids at a 100 times higher concentration (AAhigh).

Diet	Sucrose	AAlow	AAhigh	Ppure
**AAlow**	**0.030**	−	−	−
**AAhigh**	0.300	**0.005**	−	−
**Ppure**	***<0.001***	***<0.001***	***<0.001***	−
**Csuc**	**0.030**	***<0.001***	0.300	***<0.001***

**Table 2:** Median survival time (in days) of bumble bees kept in micro-colonies fed different diets, including the lower and upper 95% confidence interval (CI). Days separated by a slash represent the upper and lower median. Abbreviations as follows: Pure pollen (Ppure), casein-sucrose (Csuc), only sucrose solution (60% by mass, Sucrose), 60% sucrose solution with natural concentrations of essential amino acids and proline as can be found in nectar (AAlow), and 60% sucrose solution with amino acids at a 100 times higher concentration (AAhigh).

Diet	Median Survival Time (Days)	Lower 95% CI	Upper 95% CI
Sucrose	20	17	23
AAlow	14/15	13	18
AAhigh	20/21	16	26
Ppure	54/55	43	101
Csuc	21/22	18	33

## Supplementary Materials

The following are available online at https://www.mdpi.com/2075-4450/11/4/247/s1, Table S1: Concentrations for ten essential amino acids, proline and other non-essential proteinogenic amino acids as found in the pollen mix used in the experiments. Table S2: Average concentrations for ten essential amino acids and proline as found in floral nectar of eight Fabaceae.
