# Supplementary material for: Adding Amino Acids to a Sucrose Diet Is Not Sufficient to Support Longevity of Adult Bumble Bees
Source: Insects. 2020 Apr 15;11(4):247. doi: 10.3390/insects11040247 (PMC7240467; doi:10.3390/insects11040247)
Supplement: Supplementary file 1 [file insects-11-00247-s001.pdf]

Supplementary Material

Table S1. Concentrations for ten essential amino acids, proline and other non-essential proteinogenic amino acids as found in the pollen mix used in the experiments, measured via ion exchange chromatography (IEC) [36,37,62]. * Tryptophan could not be measured with IEC.

Amino acid	AAlow [µg/g]
Alanine (Ala)	162.93
Arginine (Arg)	63.3
Asparagine (Asn)	0
Aspartic acid (Asp)	199.97
Cysteine (Cys)	4.18
Glutamine (Gln)	0
Glutamic acid (Glu)	163.21
Glycine (Gly)	185
Histidine (His)	33.48
Isoleucine (Iso)	40.1
Leucine (Leu)	126.78
Lysine (Lys)	105.31
Methionine (Met)	31.7
Phenylalanine (Phe)	54.77
Proline (Pro)	115.92
Serine (Ser)	143.79
Threonine (Thr)	84.83
Tryptophan (Trp)	0*
Tyrosine (Tyr)	38.72
Valine (Val)	54.33

Table 2. Average concentrations for ten essential amino acids and proline as found in floral nectar of eight Fabaceae (*Trifolium hybridum, T. pratense, T. repens, T. campestre, Medicago varia, Onobrychis vicifolia, Vicia cracca* and *Lotus corniculatus*). Data from Venjakob, Klein and Leonhardt (unpublished data). *We used the average concentration of all other amino acids for tryptophan, which could not be measured with ion exchange chromatography (IEC).

Amino acid	AAlow [µg/ml]
Arginine (Arg)	2.04
Histidine (His)	1.82
Isoleucine (Iso)	2.06
Leucine (Leu)	2.63
Lysine (Lys)	2.14
Methionine (Met)	3.56
Phenylalanine (Phe)	4.39
Proline (Pro)	1.88
Threonine (Thr)	2.52
Tryptophan (Trp)	2.59*
Valine (Val)	2.89
